# High frequency of *Plasmodium falciparum *chloroquine resistance marker (*pfcrt *T76 mutation) in Yemen: An urgent need to re-examine malaria drug policy

**DOI:** 10.1186/1756-3305-4-94

**Published:** 2011-05-27

**Authors:** Abdulsalam M Al-Mekhlafi, Mohammed AK Mahdy, Hesham M Al-Mekhlafi, Ahmed A Azazy, Mun Yik Fong

**Affiliations:** 1 Department of Parasitology, Faculty of Medicine, University of Malaya, 50603 Kuala Lumpur, Malaysia; 2 Department of Parasitology, Faculty of Medicine and Health Sciences, Sana'a University, Sana'a, Yemen

## Abstract

**Background:**

Malaria remains a significant health problem in Yemen with *Plasmodium falciparum *being the predominant species which is responsible for 90% of the malaria cases. Despite serious concerns regarding increasing drug resistance, chloroquine is still used for the prevention and treatment of malaria in Yemen. This study was carried out to determine the prevalence of choloroquine resistance (CQR) of *P. falciparum *isolated from Yemen based on the *pfcrt *T76 mutation.

**Methods:**

A cross-sectional study was carried out among 511 participants from four governorates in Yemen. Blood samples were screened using microscopic and species-specific nested PCR based on the 18S rRNA gene to detect and identify *Plasmodium *species. Blood samples positive for *P. falciparum *were used for detecting the *pfcrt *T76 mutation using nested-PCR.

**Results:**

The prevalence of *pfcrt *T76 mutation was 81.5% (66 of 81 isolates). Coastal areas/foothills had higher prevalence of *pfcrt *T76 mutation compared to highland areas (90.5% *vs *71.8%) (p = 0.031). The *pfcrt *T76 mutation had a significant association with parasitaemia (p = 0.045). Univariate analysis shows a significant association of *pfcrt *T76 mutation with people aged > 10 years (OR = 9, 95% CI = 2.3 - 36.2, p = 0.001), low household income (OR = 5, 95% CI = 1.3 - 19.5, p = 0.027), no insecticide spray (OR = 3.7, 95% CI = 1.16 - 11.86, p = 0.025) and not sleeping under insecticide treated nets (ITNs) (OR = 4.8, 95% CI = 1.38 - 16.78, p = 0.01). Logistic regression model confirmed age > 10 years and low household income as predictors of *pfcrt *T76 mutation in Yemen *P. falciparum *isolates.

**Conclusions:**

The high prevalence of *pfcrt *T76 mutation in Yemen could be a predictive marker for the prevalence of *P. falciparum *CQR. This finding shows the necessity for an in-vivo therapeutic efficacy test for CQ.* P. falciparum *CQR should be addressed in the national strategy to control malaria.

## Background

Malaria remains the major cause of disease and death in the world, especially among children and pregnant women [1-4]. Malaria treatment depends on the type and severity of the disease. Chloroquine (CQ) is safe, inexpensive, and used for the treatment of uncomplicated malaria [5]. However, since the early 1960s, resistance of *P. falciparum *to chloroquine has been rising [6-10]. The *P. falciparum *CQ resistance transporter (*pfcrt*) T76 mutation is the most important molecular marker [11-18]. This point of mutation has shown strong correlation with *in vivo *and *in vitro *CQ resistance making *pfcrt *T76 mutation a significant marker for the prediction of the spread of *P. falciparum *CQ resistance [7,16,17,19-23]

In Yemen, where 60% of the total population live in malarious areas, the disease remains a significant health problem but chloroquine is still used [10,24]. Three *Plasmodium *species (*P. falciparum, P. vivax *and *P. malariae*) are reported in Yemen with *P. falciparum *being the predominant species [24-29]. The CQR problem is constantly growing since the detection of the first indigenous cases of *P. falciparum *CQR in 1989 [24]. Over the past two decades, few studies have been carried out to examine antimalarial drug efficacies in Yemen, both *in vivo *and *in vitro *[24,25,30]. However, no information about antimalarial drug resistance is available from highland areas which are endemic for malaria in Yemen. This study is the first study which aims to determine the prevalence of *pfcrt *76T mutation among patients with *P. falciparum *in Yemen and to compare the prevalence of *pfcrt *76T mutation between highland and coastal areas/foothills.

## Materials and methods

### Study areas and population

**Figure 1:**
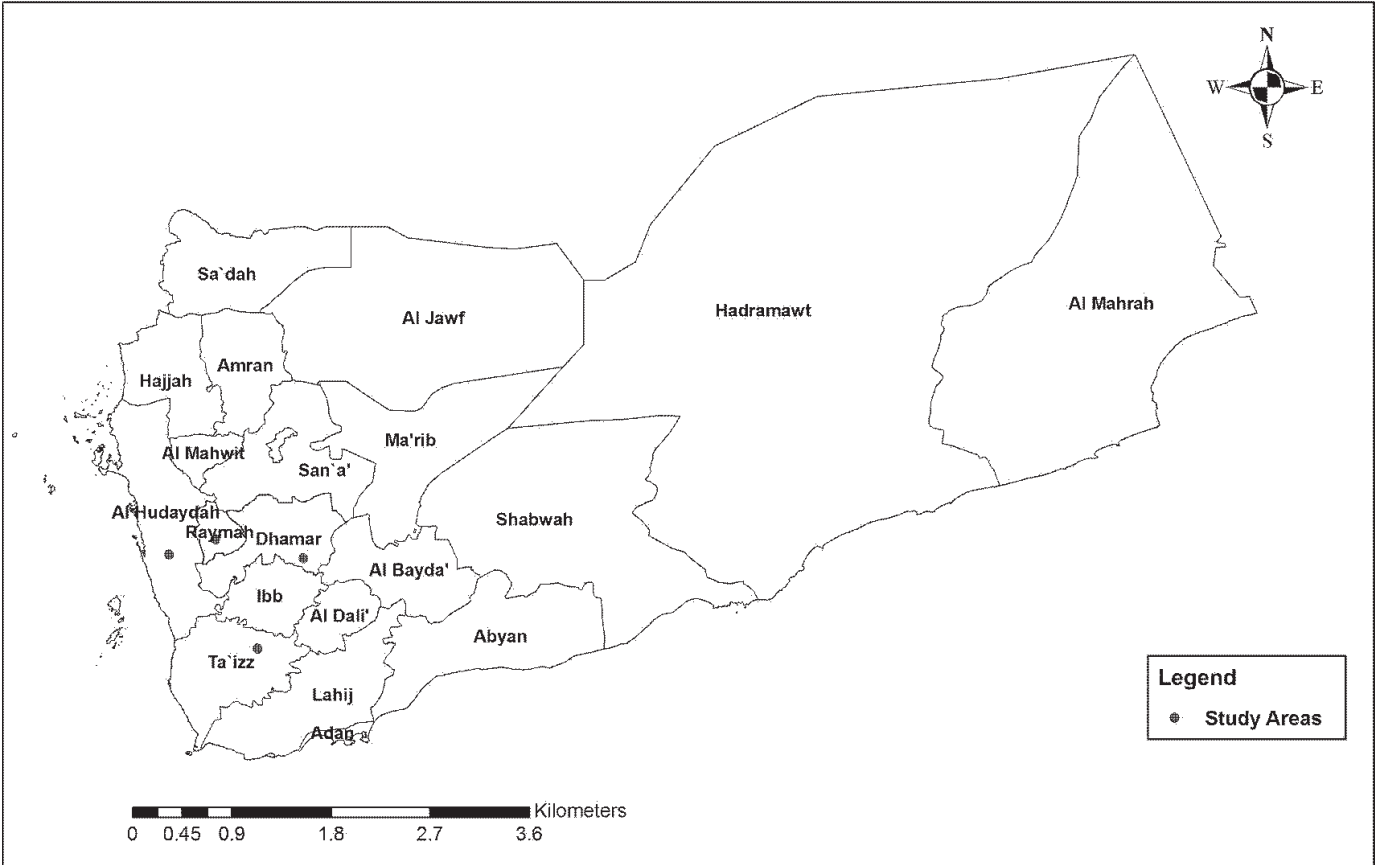
**A geographic map shows study area**.

The present study was conducted in four governorates of Yemen (Figure 1). Taiz and Hodeidah represent the mountainous hinterland and coastal areas, respectively. Rymah and Dhamar represent the highland areas. The peak time of malaria transmission in the coastal areas occurs in winter (October-April), while in the western mountains, the peak occurs in the summer (May-September). In the highlands areas (> 2000 metres above sea level), the transmission of malaria occurs throughout the year [31]*. Anopheles arabiensis *is the main vector in the country but *A. culicifacies *plays an important role in the transmission of malaria in the coastal areas. *A. sergenti *has also been reported in the mountainous hinterland and highland areas [31]. In the western costal area, temperatures are very high and occasionally exceed 54°C with irregular heavy torrents of rainfall. The mountainous hinterland receives from about 1,000-1,500 millimetres of rain each year with mild temperature in summer and cold in the winter with average temperature of 21°C. The climate in the highlands is characterized by a temperate, rainy summer with an average temperature of 20°C. The minimum sample size required for this study (246 subjects) was calculated based on previous prevalence reported in Yemen with 5% significance level and confidence level of 95% [24,27,32].

### Data collection

A total of 511 blood samples were collected between June 2008 and March 2009. Blood samples were collected through finger pricks from febrile patients attending public and private health centres and hospitals. Thin and thick blood films were prepared and blood drops were spotted on Whatman filter papers 3 MM (Whatman International Ltd., Maidstone, England). Demographic and socioeconomic information of participants were collected using a pre-tested questionnaire.

### Microscopic examination

Thick and thin blood films were prepared, air dried and stained with diluted Giemsa stain (1:20, vol/vol) for 20 min. All slides were examined by local microscopists for malaria parasites. Re-examination and species level identification were performed in a blind manner, in two different laboratories by two expert microscopists following standard and quality-controlled procedures. Parasitaemia levels were obtained from thick smears by counting the number of asexual parasites against 200 leucocytes and expressed per micro liter of blood using an assumed leukocyte count of 8000 wbc/ul.

### Molecular identification of *Plasmodium *species and *pfcrt *T76 mutation

Parasite genomic DNA was extracted from the blood collected on filter paper. DNA was extracted using QIAgen DNA Mini Kit for Blood and Tissue (QIAGEN, Germany) according to the manufacturer's instructions. Nested PCR assays based on 18S rRNA gene were used to detect and identify the *Plasmodium *species as mentioned previously [33]. The detection of the *pfcrt *T76 mutation was done according to the protocol described previously [7,34]. The allele-specific polymerase chain reaction (AS-PCR) was used. Nest 1 was carried out using 250 nM of TCRP1 (5'-CCG TTA ATA ATA AAT ACA CGC AG-3') and TCRP2 (5'-CGG ATG TTA CAA AAC TAT AGT TAC C-3') primers in a 25 μl reaction mixture containing 1X PCR buffer (iNtRON), 2.5 mM MgCl_2 _(iNtRON), 0.2 mM of dNTPs (iNtRON), 1 U of Taq polymerase (iNtRON) and 4 μl of extracted DNA. Initial denaturation was carried out for 3 minutes at 94°C followed by 30 cycles of denaturing for 30 seconds at 94°C, annealing for 30 seconds at 56°C and extension at 60°C for 1 minute. The final extension was carried out at 60°C for 3 minutes.

For allele-specific PCR, 2 μl of the nest 1 product was used in a volume 25 μl reaction containing 1X PCR buffer (iNtRON), 1.5 mM MgCl_2 _(iNtRON), 0.2 mM of dNTPs (iNtRON), and 1 U of Taq polymerase (iNtRON). The primer TCRP3 (5'-TGA CGA GCG TTA TAG AG-3') was used with a mutant-specific primer TCRP4 m (5'-GTT CTT TTA GCA AAA ATT G-3') or a wild-type-specific primer TCRPw (5'-GTT CTT TTA GCA AAA ATT T-3'). The PCR conditions were performed same as for nest 1, except that the annealing was 47°C, extension was 64°C and the number of cycles were 25. The PCR products were resolved by electrophoresis in a 1.5% agarose gel.

### Data analysis

Data analysis was performed by SPSS version 11.5 (SPSS Inc., Chicago, IL, USA). Chi-square test (or Fisher's exact test when required) was used to test the differences between groups. For univariate analysis, odds ratio and 95% confidence interval were used. A value of *P *< 0.05 was considered statistically significant. Those variables that showed significance with P < 0.05, were used to develop a stepwise forward logistic regression model.

### Ethical approval

The protocol of this study was approved by Sana'a University, Republic of Yemen and participants with febrile illness who gave written informed consents were included in this study.

## Results

### Demographic characteristic

A total of 511 participants (268 males and 242 females), with a median age of 20 years and interquartile range of 22 years voluntarily participated in this study. The basic characteristics of the participants are shown in Table 1.

**Table 1:** Characteristic of study population

Variable	n	%
**Age (years)**		
≤ 10	147	29
>10	364	71
**Sex**		
Male	268	47
Female	241	53
**Residence**		
Rural	275	54
Urban	234	46
**Household income**		
High (≥ 100 US dollars/month)	187	37
Low (< 100 US dollars/month)	322	63
Quality of housing*		
Good	456	90
Bad	52	10
**Clothes**		
Long (covering arms and legs)	298	59
Short	208	41
**Insecticide spray**		
Yes	152	30
No	353	70
**Using ITNs**		
Yes	152	30
No	356	70
**Water stream **(≤ 200 m of household)		
Yes	167	33
No	340	67
**Closing house windows**		
Yes	327	64
No	181	36

n: number of subjects

*Good housing condition was defined as being complete, made up of stones/bricks with no opening or holes. Bad housing condition did not have all these characteristics

Out of 511 blood specimens, malaria parasites were detected in 78 (15.3%) by microscopic examination for the thin and thick blood films as compared to 86 (16.8%) by nested PCR amplification of the 18S rRNA gene. Majority of the malarial infections were due to *P. falciparum *(80.3%).

### Prevalence and distribution

**Figure 2:**
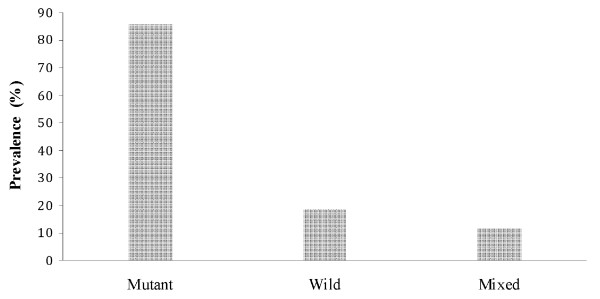
**Prevalence of *pfcrt *T76 mutation in *Plasmodium falciparum *Yemen isolates**.

**Figure 3:**
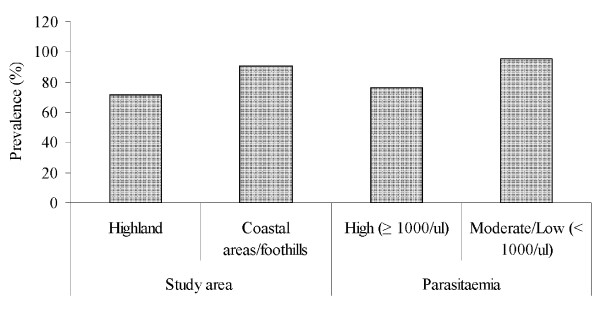
**Prevalence of *pfcrt *T76 mutation in *Plasmodium falciparum *Yemen isolates in different endemic areas and according to parasitaemia**.

Of the 81 *P. falciparum *positive samples, *pfcrt *76T mutation was detected in 66 (81.5%). Wild type was detected in 15 (18.5%) and 10 (10.3%) were mixed (Figure 2). The *pfcrt *76T mutation was detected in all governorates where the study was conducted. Prevalence was significantly higher in coastal areas/foothills (90.5%) as compared to highland areas (71.8%) (χ^2 ^= 4.67, P = 0.031). There was a significant association between *pfcrt *76T mutation and parasitaemia (Fisher exact test, P = 0.045) (Figure 3).

### Univariate and multivariate analysis

The associations of the prevalence of *pfcrt *76T mutations with the potential risk factors are presented in Table 2. The *pfcrt *76T mutation was significantly associated with age group > 10 years (OR = 9.2, 95% CI = 2.3-36.2, P = 0.001), low household income (OR = 5.0, 95% CI = 1.3-19.5, P = 0.027), not spraying insecticides (OR = 3.7, 95% CI = 1.16-11.86, P = 0.025) and not using ITNs (OR = 4.8, 95% CI = 1.38-16.78, P = 0.01). By using logistic regression analysis, the age group > 10 years and low household income were retained as significant factors associated with *pfcrt *76T mutations.

**Table 2:** Socio-economic factors associated with *pfcrt *T76 mutation

Variable	*P.f *n	*Pfcrt *T76 n (%)	OR	95% CI	*P *value^#^
**Age (years)**					
≤ 10	32	20 (63)	9.2	2.3-36.2	0.001^‡^
>10	49	46 (94)			
**Sex**					
Male	51	42 (82)	1.16	0.37-3.71	0.506
Female	30	24 (80)			
**Residence**					
Rural	66	54 (82)	1.12	0.07-4.6	0.56
Urban	15	12 (80)			
**Household income**					
High (≥ 100 US dollars/month)	11	6 (55)	5	1.3-19.5	0.027^‡^
Low (< 100 US dollars/month)	70	60 (86)			
Quality of housing*					
Good	71	57 (80)	2.21	0.25-18.92	0.408
Bad	10	9 (90)			
**Clothes**					
Long (covering arms and legs)	27	24 (89)	0.26	0.06-1.3	0.068
Short	54	42 (78)			
**Insecticide spray**					
Yes	28	19 (68)	3.7	1.16-11.86	0.025
No	53	47 (89)			
**Using ITNs**					
Yes	35	24 (69)	4.8	1.38-16.78	0.01
No	46	42 (91)			
**Water stream **(≤ 200 m of household)					
Yes	49	39 (80)	0.72	0.22-2.35	0.407
No	32	27 (84)			
**Closing house windows**					
Yes	35	28 (80)	1.18	0.38-3.66	0.492
No	46	38 (83)			

n: number of subjects

^‡ ^Confirmed as a significant risk factor by logistic regression

*Good housing condition was defined as being complete, made up of stones/bricks with no opening or holes. Bad housing condition did not have all these characteristics.

## Discussion

Malaria is an endemic disease in Yemen which causes thousands of deaths per year and almost two-thirds of the people are at risk of acquiring the disease. Yemen has not achieved significant decrease in the annual number of malaria cases as compared to other countries in the region such as Iraq, Iran and Saudi Arabia which have entered the malaria elimination or pre-elimination stage [10]. *P. falciparum *is the predominant species in Yemen, accounted for about 90% of malaria cases [24,25,27,28,31,35]. This study depends on the detection of a single point mutation in codon 76T of the *pfcrt *gene, which has shown a strong and significant association with *in vitro *and *in vivo *susceptibility to chloroquine [11-18].

The study showed high prevalence of *pfcrt *76T mutation in Yemen with coastal areas/foothills having higher rate as compared to highland areas. This finding is consistent with previous *in vivo *and *in vitro *studies carried out in coastal areas/foothills [24,30]. In Hodeidah (coastland areas), *in vivo *study found CQR in 71% of *P. falciparum*-infected patients[25]. In *in vitro *study, carried out in the same area using mark III technique, reported 47% of CQR [30]. CQR was also reported from Taiz (foothills areas) using a 7-day *in vivo *test [24]. However, there are no previous reports of CQR from highland areas endemic for malaria in Yemen. The *pfcrt *76T mutation has been considered as a key determinant of chloroquine treatment failure both *in vitro *and in the field [11-18]. Thus, the high prevalence of this marker warrants public health awareness on the emergence and spread of *P. falciparum *CQR in Yemen.

The present study found significant association of *pfcrt *76T mutation with moderate/low parasitaemia. The relationship between *pfcrt *76T mutation and malaria severity is a controversial issue. Studies carried out in Sudan [36] and Congo [37] showed no significant association between severe malaria and frequency of *pfcrt *76T mutation. However, significant association of *pfcrt *76T mutation with severity of malaria was reported from India [38] and Mali [21]. The reason for these observations is still unclear [21].

Logistic regression analysis of socio-economic factors indicated a significant association of *pfcrt *76T mutation with the age group > 10 years and low household income. These findings may be explained by the fact that these factors may increase human exposure to mosquito bites leading to high intensity of malaria transmission. Adults, especially in the coastal areas/foothill during the hot season, wear short clothes and may sleep in the courtyard of the house. Low household income plays a significant role in malaria transmission since it affects housing, living place, prevention and treatment [39-41]. The correlation between intensity of malaria transmission and genetic diversity, which may provide a mechanism for the emergence of drug resistance in *P. falciparum *population, has been reported [42].

## Conclusions

Our study reveals high prevalence of *pfcrt *76T point mutation in Yemen, suggesting high prevalence and spread of *P. falciparum *CQR. This should be taken into consideration in the national strategy to control malaria. Data from this study support an urgent need to re-examine malaria drug policy. Continuous surveillance to detect emergence of anti-malarial drug resistance is essential in Yemen.

## Competing interests

The authors declare that they have no competing interests.

## Authors' contributions

AMA, MAKM, FMY and AAA designed the study; AMA the field study, carried out the laboratory work and collated the data; AMA, MAKM and HMA performed the statistical analysis; AMA, MAKM, FMY and AAA interpreted the data; AMA and MAKM drafted the manuscript; FMY, AAA, and HMA contributed to the revision of the manuscript. All authors read and approved the final manuscript.
